# Biomolecular Mechanisms and Case Series Study of Socket Preservation with Tooth Grafts

**DOI:** 10.3390/jcm12175611

**Published:** 2023-08-28

**Authors:** Elio Minetti, Gianna Dipalma, Andrea Palermo, Assunta Patano, Alessio Danilo Inchingolo, Angelo Michele Inchingolo, Francesco Inchingolo

**Affiliations:** 1 Department of Biomedical, Surgical, Dental Science, University of Milan, 20161 Milan, Italy; elio.minetti@unimi.it; 2 Department of Interdisciplinary Medicine, University of Bari “Aldo Moro”, 70124 Bari, Italy; giannadipalma@tiscali.it (G.D.); assuntapatano@gmail.com (A.P.); ad.inchingolo@libero.it (A.D.I.); 3 College of Medicine and Dentistry, Birmingham B4 6BN, UK; andrea.palermo2004@libero.it (A.P.); francesco.inchingolo@uniba.it (F.I.)

**Keywords:** autogenous dentin graft, bone regeneration, dental biomaterials, granules, socket preservation, tooth graft

## Abstract

The purpose of this research was to assess the effectiveness of an innovative medical device capable of extracting tooth graft materials directly from the patient’s own teeth. Twenty consecutive tooth grafting procedures were conducted, with an average follow-up period of 18 months. Methods: Twenty patients requiring tooth extraction underwent socket preservation utilizing the extracted tooth as the grafting material. Results: After a 4-month healing period, the defects were significantly filled with newly formed hard tissue. Subsequently, bone biopsies were performed during dental implant placement to evaluate histological outcomes. The tissue exhibited a similar density to medium-density bone, displaying a homogeneous and uniform appearance without any visible signs of inflammation. The post-operative healing phase was free from infective complications or indications of graft particles within the regenerated bone structure. The histomorphometric analyses revealed the following results: bone total volume, BV% 52.6 ± 13.09, vital bone VB% 40.39 ± 15.86, residual graft % 12.20 ± 12.34. Conclusion: The study demonstrated positive bony healing in guided regenerative surgery procedures using autologous tooth grafts. However, further research with an extended follow-up period is necessary to thoroughly assess the potential of demineralized dentin autografts.

## 1. Introduction

Graft materials have been widely used for pre and peri-implant bone augmentation they are correctprocedures for over 35 years [1,2,3]. The most commonly used graft materials are of animal, synthetic, or human origin. In these cases, bone regeneration stimulation is solely derived from the host organism and not from the donor, thus slowing down or reducing the regenerative potential [1,2,4].

Autologous bone grafting is considered the gold standard for repairing alveolar bone defects, but it is associated with possible complications and morbidity of the donor, as well as limited availability. The human dentin matrix presents itself as an exceptional alternative to autologous or heterologous bone grafts. The crucial step in the entire process remains the preparation technique employed to convert autologous teeth into a suitable grafting material. Establishing an effective regenerative protocol is vital for the restoration and long-term preservation of both hard and soft tissue dimensions. Additionally, the selection of graft material and its inherent properties significantly influences the outcomes [5,6,7,8].

The use of teeth as graft material was first proposed in 1967 when Urist et al. [5,7] demonstrated the osteoinductive properties of demineralized autologous dental matrix. The idea of using autologous teeth instead of bone in grafting procedures arose from the observation of the similar chemical composition of dentin and bone. Both are composed of 70% inorganic portion (hydroxyapatite), 18% collagen, 2% proteins, and 10% fluids. The tooth and alveolar bone originate from neural crest cells and share the same composition of type I collagen.

In 1989, Kawai and Urist [9] first discovered partially purified bone morphogenetic proteins in bovine dental matrix. In 1991, Bessho et al. [10] showed the existence of bone morphogenetic proteins (BMPs) derived from the dentin matrix of human teeth by utilizing extracted human teeth. From this information, it can be inferred that both dentin and bone matrix serve as reservoirs for growth factors such as BMPs and basic fibroblast growth factor. There have been reports indicating that the demineralized human dental matrix, in the presence of osteoblastic cells, has the ability to promote the formation of bone and cartilage in mouse muscles [11]. The substantial number of recent studies on this topic attests to the growing scientific interest in this grafting procedure [12,13,14]. The demineralized dentin matrix can serve as an efficient reservoir for BMPs because BMPs are highly soluble and do not exert osteoinductive effects when used alone, as they are rapidly washed out. Bioactive growth factors (GFs), such as transforming growth factor-B (TGF-β) and BMPs, are present in dentin and are implicated in bone repair treatments [14]. After nearly 50 years of research, it is now possible to use autologous teeth as a substrate to produce graft material. The crucial stage in the overall procedure is the technique used to prepare autologous teeth as the graft material. Preserving the organic autologous components is vital to stimulate bone progenitor cells, while removing contaminants is necessary to prevent inflammatory or infectious reactions. Additionally, preparing the inorganic part ensures easy colonization by osteoblasts. The demineralization process is required to facilitate the release of growth factors and proteins, as the presence of non-resorbable hydroxyapatite crystals can sometimes hinder their release [15,16,17].

The resorption of dental structure allows for the release of autologous growth factors contained within the tooth itself. Demineralization facilitates the release of growth factors from the dental matrix by reducing the mineral phase [18]. Currently, demineralized autologous dentin is available in two forms: granules and blocks [19]. Some authors have supposed that the geometry and size of the granules play a fundamental role in terms of bone regeneration properties [15,20].

Recently, in a comparative test of various granule sizes and three levels of demineralization, Koga et al. recommended the use of 1000 μm particles and partial demineralization [21].

There are certain devices available on the market that enable the grinding of a patient’s tooth to generate dentin granules. However, these devices lack automated controls for physicians during the grinding process, resulting in limited control over the particle size. Additionally, the chemical treatment to achieve decontamination and demineralization of the teeth relies on the manual skills of the operator [22]. Recently, an innovative medical device (TT TOOTH TRANSFORMER^®^ SRL, Via Washington, 59—Milan, Italy) has been introduced to the market for obtaining suitable dental graft materials from the entire patient’s tooth. All grinding and demineralization processes are fully automated without the risk of human error. As per the manufacturer’s claims, this innovative device is considered an advanced system in the field of tissue engineering. It has the capability to efficiently process and convert extracted teeth into a valuable bone graft material within a short timeframe.

The autogenous genetic content ensures absolute compatibility with the recipient site, and, most importantly, the presence of morphogenetic proteins, particularly BMP-2 (a bone morphogenetic protein that stimulates bone growth), should guarantee high osteoinduction.

This autologous dental graft should be able to stimulate cell adhesion, proliferation, and differentiation, and promote bone regeneration. The treatment of dentin increases the size of dentinal tubules, further enhancing its already high wettability, which allows for easy manipulation and improved cell adhesion, as observed in Koga’s tests [21]. As for bone, the tooth consists of hydroxyapatite (HA) minerals and proteins that can be divided into collagenous (type I collagen) and non-collagenous proteins (growth factors). The Tooth Transformer reduces the crystallinity of HA, eliminates bacteria, and transforms dentin into an autologous graft material. In vitro studies have demonstrated the presence and release of BMP-2 from the tooth and the high biocompatibility of dentin after treatment [23,24]. This article aims to present the clinical and histological results of 25 socket preservation cases using the tooth, treated with the TT, as a graft material.

## 2. Materials and Methods

The study was conducted following the guidelines set forth by the University of Chieti Ethics Committee. The clinical study protocol was authorized on 21 March 2019 and registered under the number 638—21/3/19.

Twenty patients requiring the extraction of the lower molar underwent socket preservation, utilizing the extracted tooth as a grafting material. After tooth extraction, the patient’s tooth was treated as follows: the removal of tartar residue using a piezoelectric instrument, the cleaning of the root surface using diamond burs, and the removal of any filling materials (gutta-percha, composite, etc.).

Finally, the tooth was divided into smaller sections to facilitate the grinding process. These tooth pieces were subsequently placed inside the grinder. The device included a disposable single-use vial, which was correctly positioned using the provided arrows. The manufacturer claims that these measures ensure the optimal release of BMP-2 and collagen, as well as complete decontamination of the tooth root. Once all the components were inserted and the device cover was closed, the main button was pressed to start the machine. The demineralized dentin graft was produced within a time frame of 30–45 min and could then be placed inside the patient’s oral cavity (TT TOOTH TRANSFORMER SRL, Via Washington, 59—Milan, Italy). This case series involved 20 patients, 12 males and 8 females, in the age range from 32 to 73 years (average age: 57.33 ± 11.09). All patients were in good health and non-smokers.

**Figure 1:**
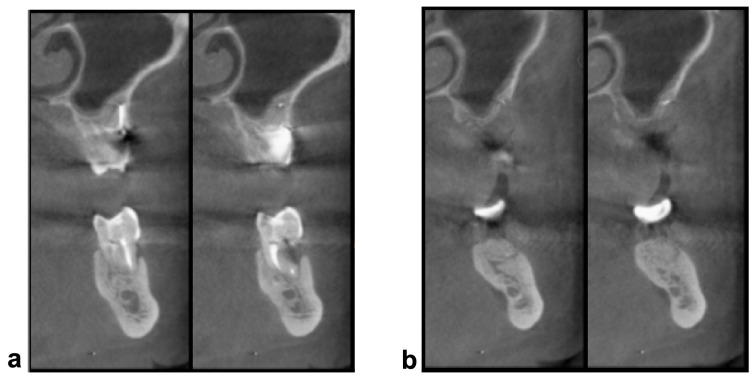
(**a**) CBCT scans acquired before grafting procedures; (**b**) CBCT scans acquired after 4 months.

Before performing tooth extraction and/or regeneration procedures, each patient underwent 3D radiological analysis. The extracted tooth was meticulously cleaned, and all foreign materials such as tartar, restorations, and endodontic filling materials were removed. After thorough drying, the tooth was inserted into the device. In all cases, a resorbable osseoguard membrane (Zimmer) was applied to cover the graft. Immediate postoperative radiological control was conducted, and each patient received clinical examinations after 10 and 30 days to assess the healing process. Cone Beam Computed Tomography (CBCT) scans were performed before the grafting procedures and after 4 months of healing to evaluate the quality and quantity of newly formed bone (Figure 1).

Histological sampling was performed during the implant placement procedure after 4 months. The bone samples were obtained when the implant was being placed. Once the patient had consented, a 3 mm trephine bur (MEISINGER USA, L.L.C. 10150 E. Easter Avenue Centennial, CO 80112, USA) was utilized to prepare the implant site. The specialized implant drills were then employed with ample irrigation using a saline solution. The bone that was extracted during the creation of a surgical implant socket was collected. Subsequently, the sample was rinsed meticulously with a physiological solution to eliminate any traces of blood or other tissue fragments. It was promptly placed into a freshly prepared fixative solution and stored in a light-protected container (10% neutral-buffered formalin) with a hermetic seal, ensuring a volume of at least 10 cc without any bubbles. During the surgical reentry, 20 titanium dental implants were placed. The average follow-up period was 12 months.

**Figure 2:**
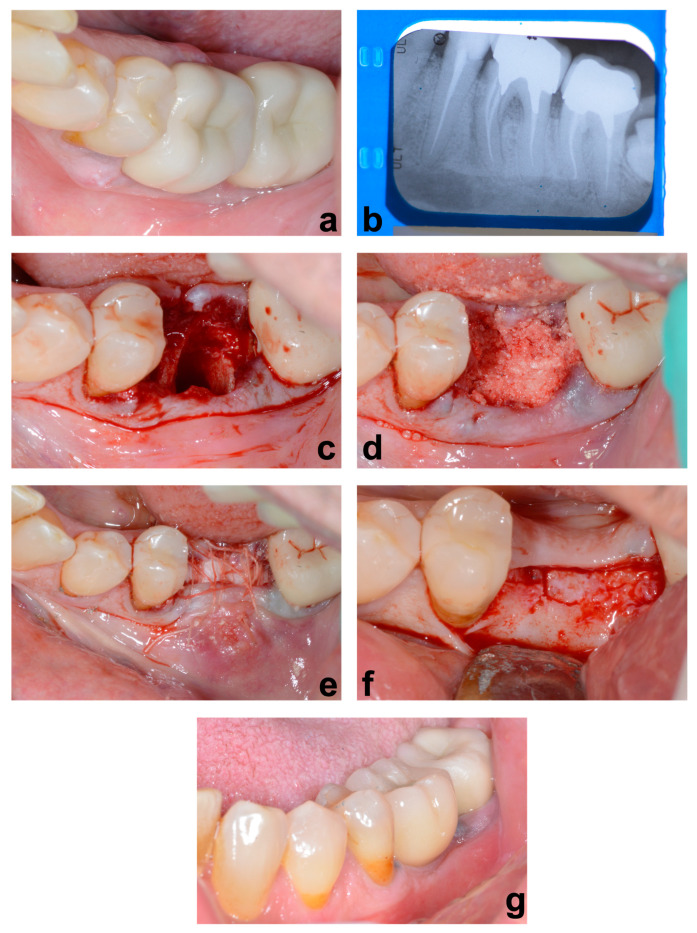
(**a**) Fracture of the mesial root of tooth 36 with vestibular wall loss; (**b**) X-ray of tooth 36 showing radicular fracture; (**c**) extraction site with bone defect and alveolar cleaning; (**d**) condensation of the treated tooth with the tooth transformer inside the socket; (**e**) suturing and coverage of dental particle using a collagen membrane; (**f**) reopening after 4 months showing the quality of the bone tissue and histological sample at the implant preparation site; (**g**) ceramic prosthesis in place.

Figure 2 shows the steps of socket preservation, utilizing the extracted tooth as grafting material.

### Histological Analysis

**Figure 3:**
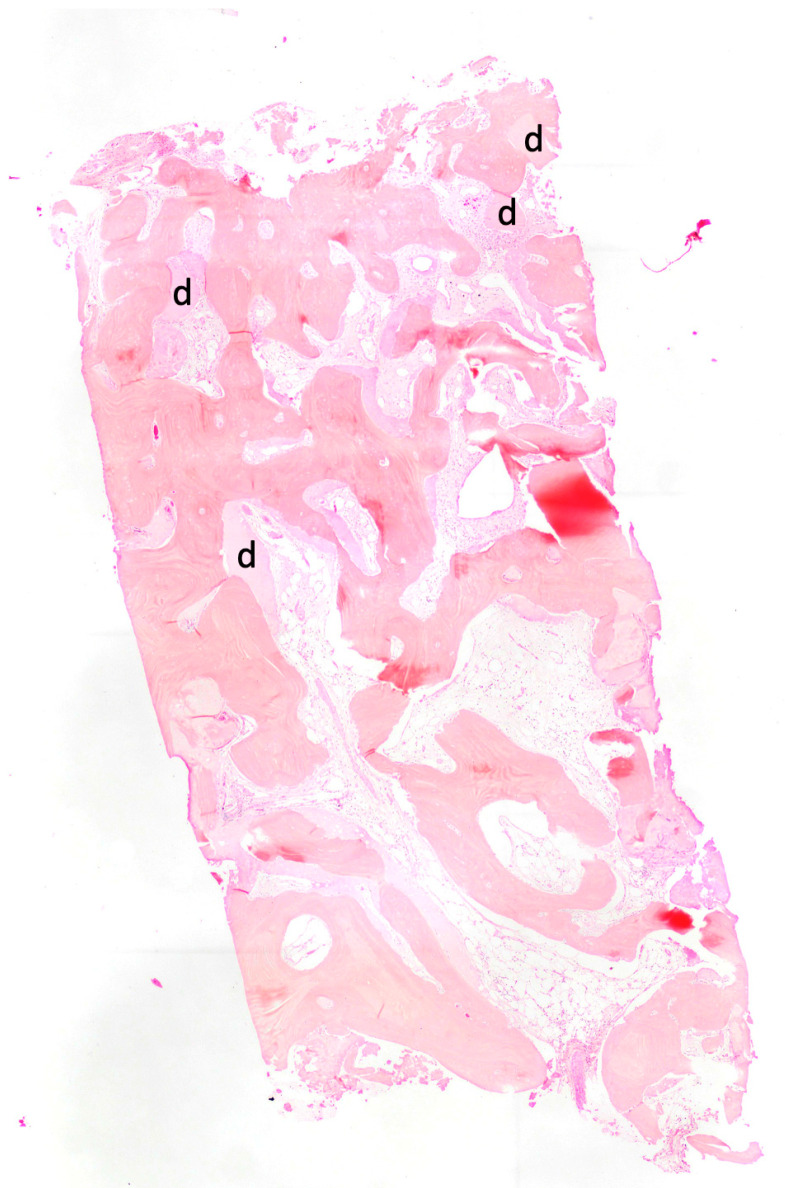
Histology of a sample, marked with “d”, showing residual dentin granules.

All samples were washed, dehydrated with increasing concentration alcohol solutions (Sigma-Aldrich, St. Louis, MO, USA), and then infiltrated into methacrylic resin (Sigma-Aldrich, St. Louis, MO, USA) for the histological analysis. Subsequently, the sample underwent processing to obtain non-decalcified sections using the LS2 disk abrasion system (Remet, Bologna, Italy) and the Micromet diamond disk cutting system (Remet, Bologna, Italy), resulting in sample slides approximately 200 microns thick. Afterwards, all samples were treated with low-abrasive paper on a lapping machine (Bueheler, Lake Bluff, IL, USA) with thickness control, gradually reducing the sample thickness to around 40–50 microns. The specimens were then polished, stained with basic fuchsin and toluidine blue, and examined using light and polarized light microscopy (Olympus, Shinjuku, Tokyo, Japan). Histological images obtained from the transmitted light microscope (Olympus, Shinjuku, Tokyo, Japan) were digitized using a digital camera and analyzed with image analysis software, IAS 2000 (QEA, Billerica, MA, USA). For each sample, the percentage of residual bone volume excluding medullary tissues (BV%), the percentage of remaining graft excluding bone and marrow (Graft%), and the percentage of vital bone excluding the medulla and residual graft (BV%) were measured and recorded (Figure 3).

## 3. Results

In total, 20 subjects (12 men and 8 women) with an average age of 57.33 years (±11.09) were enrolled for the research. Overall, 20 teeth were extracted and utilized for alveolar socket preservation therapy. In all cases, after 4 months of healing after all surgery treatments, no complications occurred and the defects were completely filled with newly formed bone. All cases achieved complete bone filling as determined by clinical and radiographic observation. The newly formed tissue observed during the surgical reentry exhibited a density comparable to medium-density bone, without the presence of graft particles or granules in the submucosal connective tissues. The regenerated bone structure appeared homogeneous and uniform, devoid of any visible graft particles or granules. The bone density detected during implant drilling procedures ranged from D2 to D3, ensuring a high level of primary stability for all inserted implants. Following appropriate healing, complete osseointegration of the implants was achieved. Over the course of the 12-month follow-up period, both the hard and soft tissues remained stable, with a notable absence of complications in soft tissue healing after the grafting procedures. Even in cases in which primary wound closure was not achieved, complete defect coverage was observed within 10–15 days, accompanied by a lack of complications or painful symptoms. After a 4-month period of healing following dental implant placement surgery, bone biopsies were collected for histomorphometric analysis. The analysis of the specimens revealed an average bone volume (BV) value of 52.6% (±13.09). The average rate of residual graft (RG) was determined to be 12.20% (±12.34), while new bone (NB) accounted for 40.39% (±15.86) of the samples. No inflammation signs were detected in all specimens. No signs of inflammation, necrosis, or filling of endodontic materials were observed in any of the specimens. Dentin and residual enamel matrices were present in all samples.

## 4. Discussion

The healing processes of post-extraction sites following bone grafting procedures have been extensively investigated through numerous clinical trials examining various graft materials [25,26,27].

Recent systematic reviews and meta-analyses have revealed the intriguing overall performance of xenogenic materials. In terms of residual graft material, procedures utilizing allografts demonstrated the lowest rates (12.4–21.11%), whereas those involving xenografts and alloplasts displayed higher results at 7 months (37.14% and 37.23%) [28].

From a clinical perspective, it has been established that when xenogenic grafts are sealed with a collagen membrane, they can effectively reduce the three-dimensional shrinkage of the bony crest. However, from a histological standpoint, these materials may undergo incomplete resorption over the long term due to their production process. The optimal graft material should possess two essential characteristics: it should serve as a scaffold for bone regeneration (osteoconduction) while also stimulating the recruitment of bone-forming cells (such as preosteoblasts) and promoting the generation of new bone (osteoinduction) [29].

In the field of bone regeneration procedures, autologous bone grafting has long been regarded as the benchmark. However, the drawbacks associated with donor site morbidity, pain, and extended hospital stays (especially when using external donor sites) have prompted the exploration of alternative bone graft substitutes [30,31]. Autologous bone grafts have been reported to resorb too quickly. Xenograft materials have been successfully used for many years in various fields of oral bone regeneration procedures, often in conjunction with dental implants [32]. Many studies have shown that these regeneration materials provide efficient scaffolding and space maintenance for the migration of osteogenic cells, but do not offer any osteoinductive properties [33,34,35].

The guided bone regeneration (GBR) healing phases are similar in the dentin and the bone because they are remarkably similar autologous tissues.

First phase:

1–4 WEEKS

The initial phase involves bleeding, inflammation, revascularization, and osteoinduction, which progress as a continuous process. Active bone production and resorption occur within four weeks of implantation. Cancellous autografts integrate with the necrotic bed by generating new bone, thereby enhancing the mechanical properties of the construct. Eventually, as the necrotic bone is resorbed and replaced, the mechanical strength of the graft–host interface is restored [36].

4–6 WEEKS

The clot forms and vascular structures migrate within the bone walls surrounding the defect. Osteoid bone deposition commences as part of this process. Cellular and molecular cascades occur, leading to the migration of cells from the surrounding tissue. These cells secrete factors crucial for bone formation and remodeling, facilitating the development of mature, remodeled bone in the underlying defect. This activation of osteoblast and osteoclast activity contributes to the process [36].

Second phase:

8–12 WEEKS

The osteoid bone matures, and the development of the cortical bone initiates. Osteoblasts mineralize the marrow bone, leading to its hardening. Additionally, new cortical bone starts to form around the outer edges [36].

Third phase:

12–16 WEEKS

The cortical bone undergoes maturation, and the process of remodeling begins for both the marrow and cortical bones. By observing near the membrane closely, one can witness the formation of newly remodeled cortical bone [36].

The chemical or physical processes used to remove any organic residues in all xenograft materials have destroyed all the proteins that are essential in promoting bone regeneration. Some authors have reported that allografts show faster regeneration and a more rapid decrease in biological activity compared to xenografts [37]. Conversely, some researchers have demonstrated that demineralized dental grafting has the ability to preserve autogenous growth factors, including osteopontin, dentin sialoprotein, and BMP. This preservation of growth factors can potentially stimulate bone formation through osteoinduction. In fact, the transplantation of dental elements frequently results in dentoalveolar ankylosis with bone replacement. These observed mechanisms may provide an explanation for how the demineralized dentin matrix acts as a slow-release vehicle for BMP after it has been resorbed [29,38,39,40]. It has been observed that dental graft material produces a similar amount of new bone as autogenous bone grafts (iliac crest) [41].

In 2014, autogenous dental bone grafting was considered a good alternative to allogeneic bone grafting when tooth extraction is required prior to surgery [42,43,44,45,46,47]. In 2016, two years following the initial publication, a case series was released documenting the long-term preservation of corticocancellous bone volume achieved through the use of autogenous tooth bone material. The average follow-up period for the cases included in the study was 5 years, demonstrating sustained success over an extended period [13].

In a recent review of the literature, 108 studies investigating the use of autogenous teeth for bone grafting were identified in total, and 6 of them were selected for analysis. The findings from these studies revealed a high implant survival rate of 97.7%, although wound dehiscence was identified as a common complication. Additionally, an animal study demonstrated that the combination of autogenous demineralized dentin matrix and polytetrafluoroethylene (PTFE) membrane led to faster bone healing compared to the use of PTFE membrane alone.

To further evaluate the effectiveness of autologous tooth as a grafting material and explore its properties and interactions with bone metabolism, a new systematic review was conducted. A comprehensive search was performed in databases including PubMed, Scopus, Cochrane Library, and Web of Science, covering articles published between 1 January 2012 and 22 November 2022. A total of 1516 studies were identified for analysis.

The review highlighted that demineralized dentin can serve as a suitable graft material due to its high compatibility with cells and its ability to promote rapid bone regeneration. It achieves an optimal balance between bone resorption and production. Additionally, using demineralized dentin as a graft material offers several advantages, such as shorter recovery times, the formation of high-quality new bone, cost-effectiveness, the elimination of disease transmission risks, outpatient procedure feasibility, and the absence of donor-related postoperative complications.

The demineralization process plays a crucial role in preparing the tooth material for grafting. It involves cleaning, grinding, and demineralization steps. Demineralization is particularly important because the presence of hydroxyapatite crystals can impede the release of growth factors, making it necessary for effective regenerative surgery [48].

In a clinical study focusing on guided bone regeneration (GBR), socket preservation, and ridge augmentation, significant new bone formation was observed. Furthermore, the amount of crestal bone resorption during the follow-up period was minimal [48,49].

Pang et al. conducted a comparative study involving 33 cases of socket preservation after tooth extraction. Their study compared the clinical and histological outcomes of using autogenous demineralized dentin matrix extracted from teeth with inorganic bovine bone. The results showed that both groups exhibited new bone formation and vertical bone gain, with no significant difference between them [50].

In 2021, a total of 504 patients from 13 dental clinics in Singapore, Spain, Czech Republic, and Italy were included in a study. Following alveolar socket preservation (ASP) procedures, 483 dental implants were successfully placed in maxillary sites. The graft material used in the ASP procedures was obtained from an innovative Tooth Transformer device, which extracted autologous demineralized tooth grafts. After a 4-month healing period, bone biopsies were conducted during placement of the dental implant to assess the histological outcomes. Following the 12-month implant loading period, the global implant survival rate, failure percentage, and peri-implant bone loss were determined. The histomorphometric analysis of the bone biopsies revealed a high percentage of bone volume (BV) at 43.58 (±12.09), along with vital new bone (NB) at 32.38 (±17.15). There were no signs of inflammation or necrosis observed. After 12 months of implant loading, only 10 dental implants experienced failure, which equated to a 2.3% failure rate and an overall implant survival rate of 98.2%. Mucositis was present in nine cases (1.8%), while peri-implantitis was observed in eight cases (1.6%). Regarding bone loss, 0.43 mm (±0.83) was detected at the mesial sites and 0.23 mm (±0.38) at the distal sites, with an average value of 0.37 mm (±0.68) (*p* > 0.568) [51].

The concept of recycling compromised teeth that require extraction, rather than discarding them, in order to avoid the use of expensive heterologous or synthetic bone substitutes, is anticipated to be well-received by patients [5].

This graft is derived from the patient’s own extracted tooth and is produced through the processing of the tooth itself using a recently introduced device. This device is capable of shredding and fully decontaminating dental materials, transforming them into a grafting material suitable for treating various types of bone defects in oral surgery procedures. The process, which involves cleaning and cutting the tooth (the duration depends on the condition of the tooth) and subsequent processing (approximately 25 min), yields approximately 0.5 to 3 g of material, depending on the tooth. There are several notable advantages to using this material. Firstly, it is entirely autogenous, meaning it originates from the patient’s own body. Therefore, it does not necessitate an additional surgical site for harvesting bone grafts. Furthermore, the structure and composition of dentin closely resemble that of bone [52].

Additionally, the material contains BMP-2, which is made available through the demineralization process, thereby providing the material with osteoinductive properties in addition to its osteoconductive features conferred by the porous three-dimensional matrix [7,10]. Another advantage is the possibility of storing the extracted teeth for an extended period of time prior to the surgery [41,53].

The findings from this study corroborate the results reported by Kim YK et al., who conducted a case series study involving 15 patients with a follow-up period of 31 months. Kim YK et al. also observed favorable bone healing through osteoconduction.

## 5. Conclusions

The findings of the current study demonstrated a significant increase in three-dimensional bone volume and a substantial percentage of vital bone formation in all socket preservation sites.

The use of the innovative grinding device enables the rapid processing and utilization of a patient’s own tooth as a bone graft. All processes of decontamination, disinfection, and demineralization are fully electronically managed by the grinding machine itself, with no possibility of error or human injury. Further studies with long-term follow-up are needed to better evaluate the potential of demineralized dentin autografts. This histological results are in line with the previous studies [39,54].

## Data Availability

Not applicable.

## Abbreviations

bone morphogenetic proteins	(BMPs)
transforming growth factor-B	(TGF-B)
polytetra-fluorethylene	(PTFE)
growth factors	(GFs)
hydroxyapatite	(HA)
Cone Beam Computerized Tomography	(CBCT)
bone volume	(BV)
residual graft	(RG)
new bone	(NB)
insulin-like growth factor	(IGF)
guided bone regeneration	(GBR)
